# Evaluation of 19 years of international external proficiency testing for high-resolution HLA typing

**DOI:** 10.3389/fgene.2023.1290915

**Published:** 2024-01-29

**Authors:** C. E. M. Voorter, L. Groeneveld, S. Heidt, L. Wieten

**Affiliations:** ^1^ Department of Transplantation Immunology, Tissue Typing Laboratory, Maastricht University Medical Center, Maastricht, Netherlands; ^2^ Department of Immunology, Leiden University Medical Center, Leiden, Netherlands; ^3^ Eurotransplant Reference Laboratory, Leiden University Medical Center, Leiden, Netherlands

**Keywords:** histocompatibility, immunogenetic tests, quality control, HLA high-resolution typing, sequencing, NGS, quality assessment, accreditation

## Abstract

The international high-resolution external proficiency testing (EPT) started in 2004 with high-resolution typing of human leucocyte antigen (HLA) class I (HLA-A,B,C) and HLA class II (HLA-DRB1, DRB345, DQB1, and DPB1) alleles, since possibilities for such an EPT within Europe were limited and all existing EPTs at that time made use of the comparison of HLA typing results without a reference. This EPT was set up as a collaboration between the HLA laboratory of Leiden, providing DNA samples to the participants, and the laboratory of Maastricht, performing the high-resolution typing as the reference result and evaluating the results of all participants according to the prevailing European Federation for Immunogenetics (EFI) standards. Once a year, 12 samples were sent to the participating laboratories, and evaluation and certificates were provided at the end of that same year. During the years, the EPT was extended to low-resolution HLA class I and II typing, high-resolution typing including DQA1 and DPA1, and allelic resolution typing for HLA class I, the latter one being unique in this field. Evaluation of the high-resolution typing results of the last 19 years showed a clear increase in the number of loci tested by the participating laboratories and a clear change of method from Sanger sequencing with additional other techniques (SSO/SSP) to the nowadays widely used next-generation sequencing method. By strictly using the EFI rules for high-resolution HLA typing, the participants were made aware of the ambiguities within exons 2 and 3 for class I and exon 2 for class II and the presence of null alleles even in a two-field HLA typing. There was an impressive learning curve, resulting in >98% correctly typed samples since 2017 and a 100% fulfillment of EFI rules for all laboratories for all loci submitted in the last 2 years. Overall, this EPT meets the need of an EPT for high-resolution typing for EFI accreditation.

## Introduction

Matching donor and recipient for human leucocyte antigens (HLAs) has been and still is important for patient and graft survival in solid organ and stem cell transplantation. For both kinds of transplantation, national and international exchange programs exist, and therefore, it is necessary to have a reliable HLA typing using identical nomenclature all over the world, irrespective of the HLA typing laboratory. For the use of identical HLA nomenclature worldwide, the World Health Organization (WHO) Nomenclature Committee for Factors of the HLA System was set up in 1968 having the responsibility for naming of new HLA genes, allele sequences, and their quality control (WHO Nomenclature Committee, 1968). Furthermore, in 1998, a unique specialist database was set up for sequences of the human major histocompatibility complex (MHC), nowadays known as the IPD-IMGT/HLA database, which is an important and highly appreciated resource for the HLA community (Barker et al., 2023).

The American Society of Histocompatibility and Immunogenetics (ASHI) and the European Federation for Immunogenetics (EFI) have both established an accreditation program for Histocompatibility and Immunogenetics (H&I) laboratories, and one of the aims is to ascertain accurate and correct HLA typing (Harmer et al., 2018; ASHI accreditation available at: https://www.ashi-hla.org/page/Accreditation. Accessed August 23, 2023). One of the requirements for both EFI and ASHI accreditation is adequate performance of (external) proficiency testing [(E)PT] for all techniques in use for accredited activities. Already from the start of the EFI accreditation program, performing high-resolution typing of at least DRB1 was one of the prerequisites to become accredited for the clinical accreditation category of unrelated stem cell transplantation (EFI standards 5.5; I2.210 available at: https://efi-web.org/committees/standards-committee. Accessed August 23, 2023). Nowadays, high-resolution typing of HLA-A, -B, -C, and -DRB1 is the minimum requirement for this clinical service (EFI standards 8.0, E5.3.4.3.1.2.1). Furthermore, for solid organ transplantation, high-resolution typing of both the recipient and the donor is now the preferred choice because this will ultimately facilitate the virtual cross matching that has recently been implemented by Eurotransplant.

Although several external proficiency testing (EPT) schemes on HLA typing were available in 2004, in many of them, not all HLA loci or no high-resolution typing was provided. Moreover, at that time, all of them made use of a comparison of HLA typing results of all participants and definition of the consensus based on the most frequently reported assignment. Therefore, we set up a high-resolution EPT for the HLA loci HLA-A, -B, -C, -DRB1, -DRB3/4/5, -DQB1, and -DPB1 as a collaboration between two laboratories: the laboratory of Leiden provided the samples, whereas the laboratory of Maastricht performed the high-resolution typing by hemizygous, group-specific Sanger sequence-based typing, providing the reference consensus typing (Voorter et al., 2014; Voorter et al., 2016). This EPT scheme is now running for the 20th year in a row. During these years, the EPT was extended to low-resolution HLA class I and II typing, high-resolution typing including HLA-DQA1 and -DPA1 and allelic resolution typing for HLA class I, the latter one being unique in this field. In this report, we evaluated the high-resolution typing results from the past 19 years.

## Materials and methods

For this EPT exercise, each year, 12 DNA samples were shipped to the participants by the Department of Immunology (formerly Immunohematology and Blood Transfusion) of the Leiden University Medical Center (LUMC). The amount of DNA was approximately 20 μg in a concentration of 100 ng/μL. The shipment was scheduled at the end of May, whereas the results had to be submitted before the 1st of October, giving the participants at least 4 months to collect their results. An introduction letter stating the rules of evaluation of results was sent together with the samples and predetermined forms to fill in the results obtained. Both letter and forms were also sent by e-mail, to fill in digitally and send back by mail. In this introduction letter, it was indicated that the typing analysis must be performed using an IPD-IMGT/HLA database that has been released not more than 1 year prior to the shipment of the samples (in accordance with the EFI standards) and that the database used (release number) must be reported.

The reference high-resolution typing was performed by the Department of Transplantation Immunology of the Maastricht University Medical Center (MUMC+) from 2004 till 2019 with the in-house method of hemizygous, group-specific Sanger sequencing (Voorter et al., 2014; Voorter et al., 2016) and from 2019 on by next-generation sequencing (NGS) using the AllType FASTplex kit (One Lambda, Life Technologies, Carlsbad, California, United States) in combination with sequencing on Illumina MiSeq. In case of phasing or other problems with the latter method, the previous hemizygous Sanger sequencing method was used in addition to resolve any ambiguities.

In 2004, the EPT started with high-resolution typing of the HLA loci A, B, C, DRB1, DRB3/4/5, DQB1, and DPB1. In 2007, the locus DQA1 and in 2021, the locus DPA1 were added to this EPT exercise. On request of several participants also, low-resolution typing was supported and evaluated since 2016 and allelic resolution typing for the HLA class I loci A, B, and C. For this allelic resolution typing, restrictions were set to the part of the gene that had to be analyzed as a minimum to comply with this EPT.

Evaluation of the results was carried out at the Maastricht laboratory, comparing the submitted typing of the participants with the reference HLA typing. The rule for high-resolution typing as described in the EFI standards was taken into account. From the beginning, it was strictly followed that all genotype ambiguities (i.e., ambiguities within exons 2 and 3 for class I and within exon 2 for class II) were counted as an error (error 1). In addition, not excluding the possible null alleles present within the indicated high-resolution typing result was counted as a mistake from 2010 for class II and from 2011 for class I (error 2). In the evaluation letters of 2009 and 2010, this was clearly stated with, as an example, DRB4*01:03 and A*03:01 that will both be counted as an error if the potential null alleles (DRB4*01:03:01:02N, A*03:01:01:02N) were not excluded. Other results that were counted as an error were (error 3) reporting a typing that is different from the consensus; this could be reporting an extra allele not present in the consensus, missing an allele that is present in the consensus, or reporting an allele different from the consensus and reporting an allele twice, whereas the allele was detected only once (no family results are present for these samples), and (error 4) reporting only a one-field result instead of two and the usage of incorrect nomenclature by the laboratory.

After the evaluation, an overview of the results of all participating laboratories including the reference result was provided to the participants, together with a certificate for each laboratory, clearly stating the number of samples performed for each HLA locus, the number of correct and incorrect samples, and the percentage of concordance with the reference result.

## Results

During the past 19 years, the number of participating laboratories to this international high-resolution EPT has been fluctuating between 18 and 30, always outnumbering the minimum required number of 10 as demanded by the EFI standards for EPT providers (vs. 7.3, standard 7.1; EFI standards for EPT providers available at: https://efi-web.org/fileadmin/Efi_web/Committees/EPT/EFI_EPT_Standards_for_Providers_v7-3_approved_April_2021.pdf. Accessed August 23, 2023). The participating laboratories were located in 12 different countries: Austria (two), Belgium (five), Denmark (two), France (two), Germany (twelve), Greece (one), Ireland (one), the Netherlands (five), Romania (one), Slovenia (one), Sweden (three), and Turkey (one).

**FIGURE 1:**
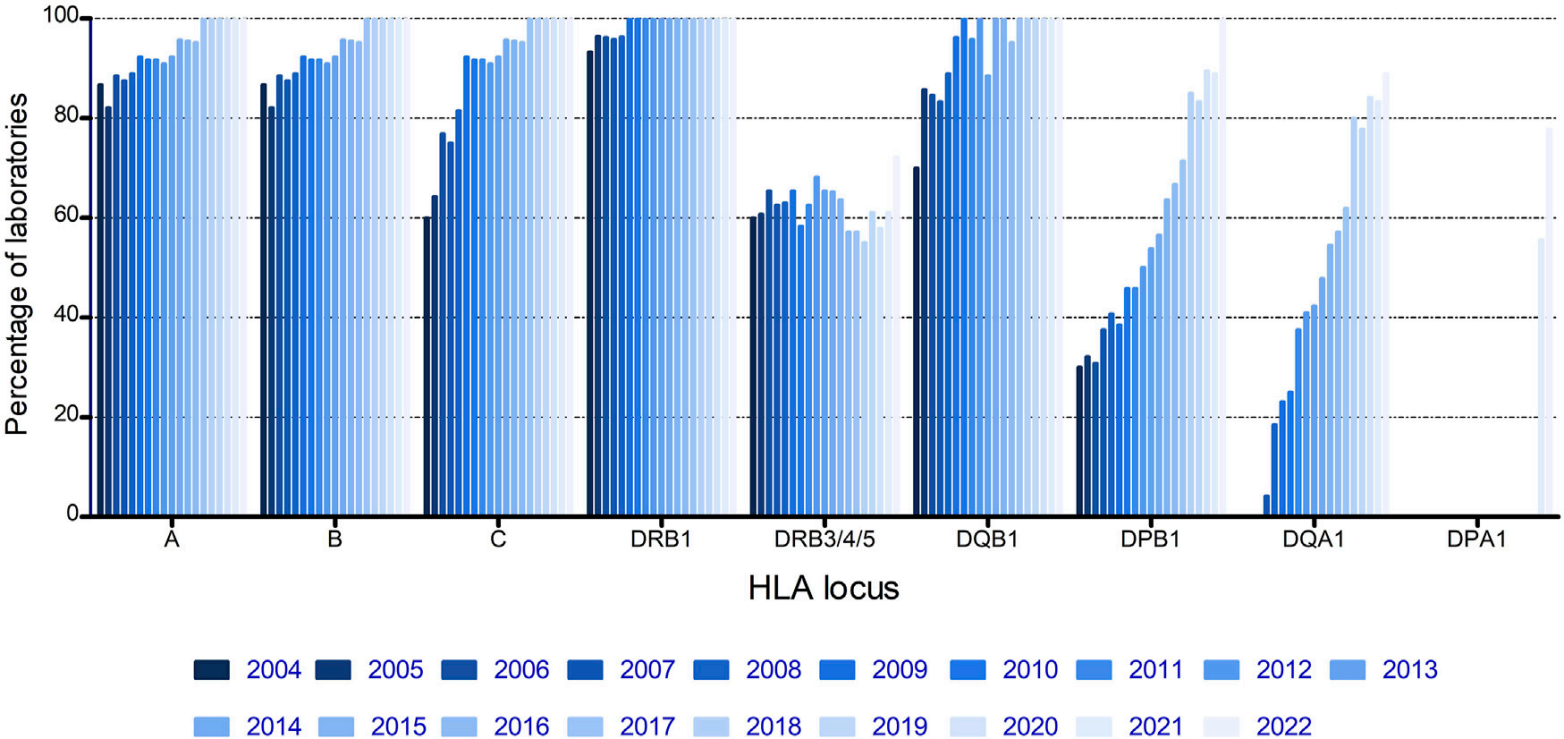
Percentage of laboratories submitting results for the HLA loci during the years.

From the start of the EPT, most laboratories submitted results for HLA-A, -B, and -DRB1, the loci that were thought to be most important at that time, and with DRB1 being mandatory for unrelated stem cell transplantation (Figure 1). Both HLA-C and -DQB1 showed a fast increase with >90% of laboratories submitting results for these loci from 2009 onwards. DPB1 and DQA1 showed a more gradual increase, whereas DRB3/4/5 showed no increase at all, with a steady 55%–70% of laboratories submitting results for these loci throughout the complete period of evaluation (Figure 1).

**FIGURE 2:**
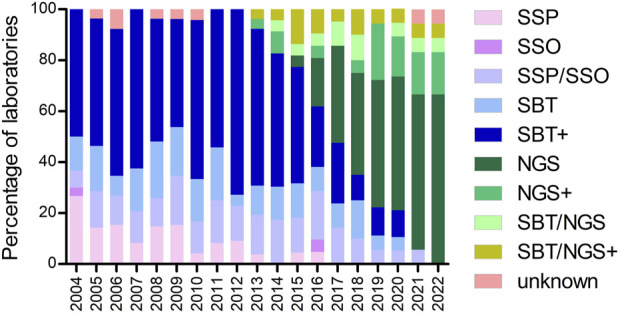
Percentage of laboratories using the methods indicated for high-resolution HLA typing during the years. Footnote: SBT+ = SBT/SSP; SBT/SSO; SBT/SSP/SSO; SBT/RLB; SBT/SSP/RLB. NGS+ = NGS/SSP; NGS/SSO; NGS/qPCR. SBT/NGS+ = SBT/NGS/SSP; SBT/NGS/SSO; SBT/NGS/SSP/SSO.

Evaluation of the methods used for high-resolution HLA typing by different laboratories demonstrates that SBT in combination with other techniques has been the prevalent method in the period from 2004 to 2016 (Figure 2). After 2016, NGS either alone or in combination with other techniques was the method of choice for the majority of the laboratories. Moreover, SSP and the combination of SSP/SSO gradually disappeared throughout the evaluation period (Figure 2).

The percentage of correctly typed samples per HLA locus and per year was calculated and is displayed in Table 1. Although the percentage of incorrectly typed samples was >20% for several loci in the initial stage of the EPT scheme, for all loci, a clear learning curve was present, resulting in >98% correctly typed samples since 2017. Notable decreases in correctly typed samples were observed in 2010 for HLA-DRB3/4/5 and in 2011 for HLA-A and -B. In those years, we started to count not excluding the null alleles as an error. In 2010, all typing results of DRB4*01:03, without mentioning that the null allele (DRB4*01:03:01:02N) was excluded, were counted as a mistake, and in 2011, the same was made for the class I results. The presence of A*01:01, A*03:01, and B*15:01, for which the null alleles (A*01:01:01:02N, A*03:01:01:02N, and B*15:01:01:02N, respectively) had to be excluded, but were not, resulted in a clear decrease in correctly typed samples in 2011, although overall 73% of the laboratories correctly excluded these null alleles.

**TABLE 1:** Percentage of samples correctly typed by all laboratories together.

Year	HLA-A	HLA-B	HLA-C	HLA-DRB1	HLA-DRB3/4/5	HLA-DQB1	HLA-DPB1	HLA-DQA1
2004	87.7	90.4	77.2	94.4	78.7	94.8	79.6	
2005	95.3	96.4	91.5	98.8	99	97.9	91.7	
2006	96.4	95.6	92.9	97.7	94.1	97	86	
2007	99.1	99.6	97	99.2	98.8	96.8	90.9	100
2008	97.5	97.8	98.4	97.7	98.4	99.6	97.5	96.9
2009	99.6	99.3	96.7	99	96.5	98.3	98.3	96.9
2010	93.8	94.6	94.6	99.3	72.2	98.9	92.9	98.6
2011	84.9	91.9	96.1	98.2	89.1	98.9	95.2	98
2012	99.6	99.1	98.3	99.6	94.8	99.6	98.4	100
2013	94.7	94.3	99.6	99.7	92.9	98.5	96.3	92.1
2014	98.5	100	99.6	100	98.9	99.6	99.4	94.6
2015	96.8	100	99.2	96.9	100	98.9	97.6	100
2016	100	99.1	99.1	99.6	94.7	100	98.1	100
2017	98.4	98.8	99.2	99.6	99.2	98.7	98.3	99.3
2018	98.7	99.6	100	99.6	98.5	99.6	99	99
2019	99.1	99.1	98.1	100	98.5	99.1	99.4	100
2020	99.1	98.2	98.7	99.6	97.7	99.1	99.5	99.5
2021	99.5	99.5	100	99.5	99.2	99.5	99.5	100
2022	99.5	100	98.9	99.5	98.6	100	99	100

**FIGURE 3:**
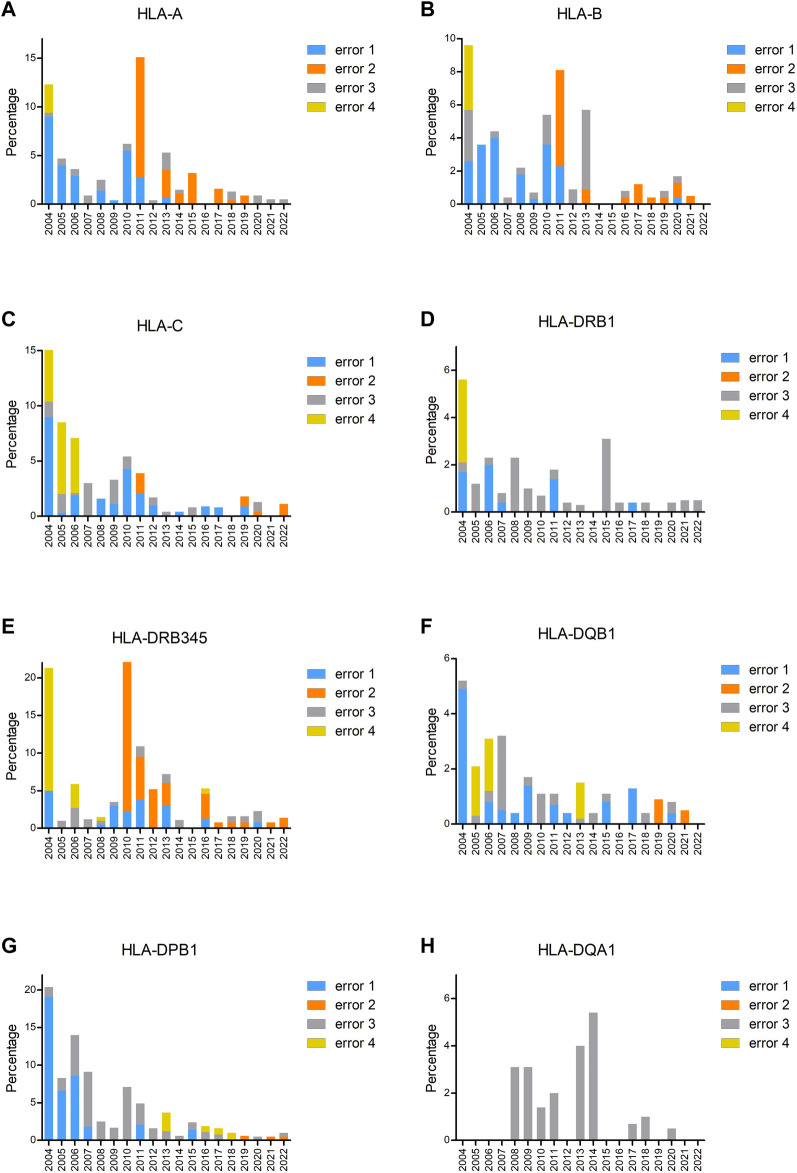
Percentage of incorrectly typed samples split up into the different types of errors for HLA-A **(A)**, HLA-B **(B)**, HLA-C **(C)**, HLA-DRB1 **(D)**, HLA-DRB3/4/5 **(E)**, HLA-DQB1 **(F)**, HLA-DPB1 **(G)**, and HLA-DQA1 **(H)**. Error 1 is genotype ambiguity (i.e., ambiguities resulting from polymorphisms located within exons 2 and 3 for HLA class I loci and exon 2 for HLA class II loci), error 2 is null alleles not excluded^A^, error 3 is incorrect allele type (i.e., allele different from consensus and missing allele or extra allele, the latter one also in case of a homozygous result), and error 4 is others (i.e., one-field typing till 2006, incorrect nomenclature from 2008)^B^. Footnote: ^A^Error 2 not excluding the null alleles concerns the following null alleles: HLA-A: *01:01:01:02N, *03:01:01:02N, *26:01:01:03N, *31:01:02:03N. HLA-B: *15:01:01:02N. HLA-C: *03:03:01:50N, *03:03:01:52N, *07:02:01:17N, *15:02:01:08N. HLA-DRB4: *01:03:01:02N, *01:03:01:13N. HLA-DPB1: *04:01:01:24N. HLA-DQB1: *03:276N. ^B^Error 4 incorrect nomenclature (from 2008 on) concerns the following: DRB3/4/5: *03:01 instead of 3*03:01; 4*01:03N instead of 4*01:03:01:02N. DQB1: *06:03/39, *06:04/41 instead of *06:03/41, *06:04/39. DPB1: *04:01/105:01, *04:02/126:01 instead of *04:01/126:01, *04:02/105:01; *02:01/105:01, *04:02/416:01 instead of *02:01/416:01, *04:02/105:01.

To investigate the type of errors that occurred for different loci, we analyzed the errors per year and per locus (Figures 3A–H). From these figures, it is clear that the increase in the incorrect results in 2011 for HLA-A and -B and in 2010 for HLA-DRB3/4/5 was due to not excluding the null alleles (error 2). An error 4 mistake at the beginning of the EPT was due to reporting of low-resolution results instead of high resolution by the participants. In later years, error 4 was primarily due to usage of incorrect nomenclature, the majority concerned incorrect reporting of allele ambiguities (i.e., ambiguities outside exons 2 and 3 for class I and outside exon 2 for class II) (see Figure 3 legends and footnote for details). A clear learning curve for resolving genotype ambiguities (error 1) could be observed for all loci. The percentage of error 3, typing an allele incorrect, is fluctuating for all the loci throughout the years, in general varying between 0% and 4% but with some outliers. For the B locus, the outlier in 2013 was due to the allele B*07:161N that was present in one of the samples, which was mistyped as B*07:02 by 55% of the laboratories. For DRB3/4/5, not only there was a 20% increase in not excluding the null alleles in 2010 but also incorrect allele typing was exceeding 4% because in one sample, the DRB4*01:03:01:02N allele was present, and this was mistyped as DRB4*01:03 by 46% of the participants. The main problem with DPB1 typing in the earlier years, up to 2010, was the incorrect reporting of an allele, without taking alleles into account with different exon 1 or exon 3 sequences that were not analyzed in the laboratory. For example, laboratories were reporting DPB1*03:01, whereas the correct allele typing was DPB1*104:01, which has an exon 2 sequence identical to DPB1*03:01, but a difference in exon 3. Reporting DPB1*03:01/104:01 would have been correct, but reporting only DPB1*03:01 is incorrect. The allele DPB1*104:01 (previously known as DPB1*0502) was included for the first time in the IPD-IMGT/HLA database of January 2005 and, therefore, had to be reported in the EPT since 2006. The outlier for DQA1 in 2014 was due to mistyping DQA1*01:01 as 01:05 and DQA1*03:03 as 03:02.

**FIGURE 4:**
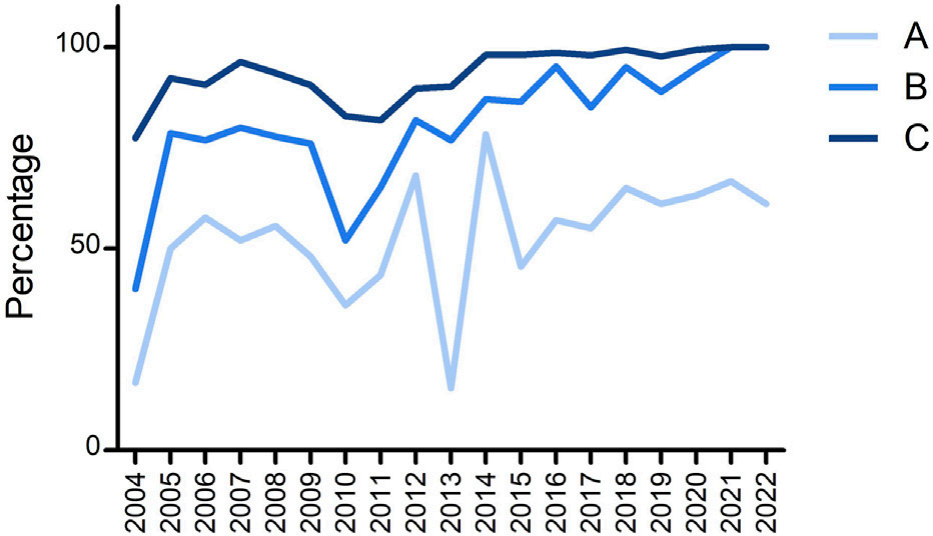
Overview of the percentage of (A) laboratories that were 100% correct for all submitted loci, (B) laboratories that fulfilled the EFI criteria for all submitted loci, and (C) submitted loci that fulfilled the EFI criteria.

Another interesting feature to check was how many laboratories had no mistakes in any of the submitted loci and whether the EFI criteria for EPT were fulfilled (i.e., >90% of results are correct). Figure 4 shows three different lines: line A shows the percentage of laboratories without any mistake in all submitted loci, line B shows the percentage of laboratories that fulfilled the EFI EPT criteria for all submitted loci, and line C shows the percentage of all submitted loci that fulfilled the EFI criteria. The steep drop in 2013 is again due to the mistyping of B*07:161N; although 45% of laboratories typed this B allele correct, some of these laboratories had another incorrect typing for a different locus. From 2014, >95% of all submitted loci fulfilled the EFI EPT criteria. In the last 2 years, all laboratories fulfilled the EFI EPT criteria for all submitted loci. Since the percentage of laboratories with 100% correct is varying here between 60% and 70%, this indicates that 30%–40% of laboratories have incorrect typing results, but maximum 1 per locus.

## Discussion

Due to the global use of HLA test results obtained by Histocompatibility and Immunogenetics (H&I) laboratories all over the world and the high clinical importance for transplantation outcome, there are strict rules for quality and accreditation requirements for these laboratories. One way of testing whether a laboratory meets these high-quality standards is by performing an external quality control for all techniques in use by the laboratory. Within Europe, there are different EPT schemes for the different EFI accreditation categories for H&I laboratories, listed on the EFI website (EFI overview of EPT providers available at: https://efi-web.org/fileadmin/Efi_web/Committees/EPT/Overview_EPT_provider_registration_January2021.pdf. Accessed August 23, 2023).

This report concerns one of the high-resolution HLA typing EPT schemes that is available within Europe. For EFI accreditation, a minimum of 10 samples is required for each molecular method and each locus. For this EPT, we decided to send 12 samples each year to anticipate any problems that might occur with a specific sample, like contamination, loss of material, or otherwise. Furthermore, in contrast to many other EPTs for HLA typing that use a consensus rule, this EPT makes use of a reference HLA typing performed by the laboratory of Maastricht. There are two major advantages of this approach, one being that the samples can always be graded, which might not be the case when the consensus rule is used and the consensus threshold is not reached. The second advantage is that if the majority of laboratories use a certain method/kit that results in an incorrect typing for a certain sample, the consensus will be the incorrect result, applying unnecessary pressure on the participants who do have the correct typing result. This was, for example, the case shortly after the discovery of the allele DPB1*104:01, which had an exon 2 sequence identical to DPB1*03:01, but a difference in exon 3. One year after this discovery, the majority of laboratories still typed DPB1*03:01, whereas the reference typing was DPB1*104:01, determined by sequencing exon 3. A potential disadvantage could be if the reference laboratory has an incorrect result, but one assumes that the reference laboratory will start evaluating their sequencing results, when all participants have a discordant result.

The different aspects that are tested with this EPT are correct sample tracking, correct HLA typing with the method used, and correct reporting of the HLA typing results. With this EPT, we are not testing the complete specimen handling, since we are providing DNA samples, whereas in most laboratories, blood or buccal swab samples will be obtained. This made the exclusion of null alleles extra challenging for the participating laboratories, since all typing information had to be obtained using a molecular method and could not be performed using serological methods, showing the presence of the molecule on the cell surface. Furthermore, from 2011, it was counted as incorrect if the null alleles were not excluded, creating awareness among the participants that even a two-field HLA result might not be a truly high-resolution typing, since there can be a null allele amongst the two-field typing results (e.g., A*03:01 can be A*03:01:01:02N). There is one exception to this rule, in case a sample is typed by full length sequencing as DQB1*03:01 homozygous, the presence of DQB1*03:276N as the second allele cannot be excluded. DQB1*03:01 and 03:276N have identical sequences from exon 2, the null allele is missing exon 1 and part of intron 1. However, for patient care, this is not a problem, since there is a DQB1*03:01 present as a molecule on the cell surface, whether the second allele is expressed or not.

The main goal of HLA typing EPTs in general is to assess the reproducibility, accuracy, and reliability of the HLA typing performed by each participating center, and as such, it contributes to high-quality level in the participating laboratories. The evaluation of all results including an error analysis is very useful for the participants to improve their diagnostic work. Therefore, we always provided an overview of all results with the errors highlighted and an explanation of the errors in detail in the accompanying letter with the intention to raise specific awareness about the presence of null alleles, the presence of genotype ambiguities, and the correct reporting of allele ambiguities.

Lin et al. (2022) described the EPT results with a national proficiency scheme from China performing HLA typing by NGS by 24 laboratories in 2021. Comparing their results with ours revealed an overall concordance rate for all HLA alleles typed of 99.2% in the China EPT and 99.5% in ours for both 2021 and 2022. The percentage of Chinese laboratories that were 100% correct for all alleles reported was 54.1%, whereas it was 66.7% in 2021, and 61.1% in 2022 in ours (see Figure 4). Apparently, although the overall performance is rather high in both EPTs, there are still a substantial number of laboratories with one or more errors in the EPT results.

Since EFI standards (vs. 6.3, effective Okt 2015; EFI standards available at: https://efi-web.org/committees/standards-committee. Accessed August 23, 2023) implemented the definition of allelic resolution, we started to assess allelic resolution typing for HLA class I in 2016, but with a very limited number of participants. According to the EFI EPT standards for providers (vs. 7.3, standard 7.2; EFI standards for EPT providers available at: https://efi-web.org/fileadmin/Efi_web/Committees/EPT/EFI_EPT_Standards_for_Providers_v7-3_approved_April_2021.pdf. Accessed August 23, 2023), it should be regarded as an EPT workshop, since the number of participants is below 10. Nevertheless, as far as the authors are aware, this is the only EPT workshop on HLA typing at the allelic resolution level. In the EFI overview of EPT providers (EFI overview of EPT providers available at: https://efi-web.org/fileadmin/Efi_web/Committees/EPT/Overview_EPT_provider_registration_January2021.pdf. Accessed August 23, 2023.) from January 2021, UK NEQAS has also indicated to provide EPT for HLA allelic resolution typing, but according to their website (UK NEQAS for H&I schemes available at: https://ukneqashandi.org.uk/schemes/. Accessed August 23, 2023), they provide HLA typing to the second and third field resolution, whereas allelic resolution is defined as a four-field typing result. The difficulty with this four-field typing is that with each update of the database, the sequence at the 5′ and/or 3’ UTR sites might have been extended, with differences between alleles located in these newly submitted sequences. No exact boundaries have been set for the HLA genes, and therefore, it is not known to what extend the gene must be sequenced to enable allelic resolution typing. For our EPT workshop on allelic resolution, we kept the boundaries of −50 and +500, implying that all ambiguities located within 50 nucleotides ahead of the start codon and 500 nucleotides after the stop codon must be resolved. These boundaries enable ongoing allelic resolution without continuous adaptation of primers, kits, and/or procedures. The participants fulfilled EFI rules for all class I loci for this allelic resolution EPT in the last 5 years.

Further improvement of this EPT will be a web-based submission in the near future in collaboration with the Eurotransplant Reference Laboratory, with upcoming possibilities to send results automatically from the laboratory information system after authorization of the results to minimize any clerical errors and to resemble the normal working flow of the laboratories.

In summary, the results of our high-resolution HLA typing EPT showed that the quality of high-resolution typing of the participants has been improved over the years, enabling EFI accreditation for all submitted loci. To keep this high quality standard, the continuing participation in external proficiency testing is of utmost importance and mandatory to be granted accreditation by the specific H&I accreditation programs of EFI and ASHI.

## Data Availability

The original contributions presented in the study are included in the article/Supplementary materials, further inquiries can be directed to the corresponding author.
